# Characterization of primary human mammary epithelial cells isolated and propagated by conditional reprogrammed cell culture

**DOI:** 10.18632/oncotarget.23817

**Published:** 2017-12-22

**Authors:** Liting Jin, Ying Qu, Liliana J. Gomez, Stacey Chung, Bingchen Han, Bowen Gao, Yong Yue, Yiping Gong, Xuefeng Liu, Farin Amersi, Catherine Dang, Armando E. Giuliano, Xiaojiang Cui

**Affiliations:** ^1^ Department of Breast Surgery, Hubei Cancer Hospital, Wuhan, 430079, China; ^2^ Department of Surgery, Samuel Oschin Comprehensive Cancer Institute, Cedars-Sinai Medical Center, Los Angeles, CA 90048, USA; ^3^ Department of Radiation, Samuel Oschin Comprehensive Cancer Institute, Cedars-Sinai Medical Center, Los Angeles, CA 90048, USA; ^4^ Department of Pathology, Lombardi Comprehensive Cancer Center, Georgetown University, Washington, DC 20057, USA

**Keywords:** conditional reprogramming, estrogen receptor, mammary epithelial cells, luminal cells, myoepithelial cells

## Abstract

**Purpose:**

Conditional reprogramming methods allow for the inexhaustible *in vitro* proliferation of primary epithelial cells from human tissue specimens. This methodology has the potential to enhance the utility of primary cell culture as a model for mammary gland research. However, few studies have systematically characterized this method in generating *in vitro* normal human mammary epithelial cell models.

**Results:**

We show that cells derived from fresh normal breast tissues can be propagated and exhibit heterogeneous morphologic features. The cultures are composed of CK18, desmoglein 3, and CK19-positive luminal cells and vimentin, p63, and CK14-positive myoepithelial cells, suggesting the maintenance of *in vivo* heterogeneity. In addition, the cultures contain subpopulations with different CD49f and EpCAM expression profiles. When grown in 3D conditions, cells self-organize into distinct structures that express either luminal or basal cell markers. Among these structures, CK8-positive cells enclosing a lumen are capable of differentiation into milk-producing cells in the presence of lactogenic stimulus. Furthermore, our short-term cultures retain the expression of ERα, as well as its ability to respond to estrogen stimulation.

**Materials and Methods:**

We have investigated conditionally reprogrammed normal epithelial cells in terms of cell type heterogeneity, cellular marker expression, and structural arrangement in two-dimensional (2D) and three-dimensional (3D) systems.

**Conclusions:**

The conditional reprogramming methodology allows generation of a heterogeneous culture from normal human mammary tissue *in vitro*. We believe that this cell culture model will provide a valuable tool to study mammary cell function and malignant transformation.

## INTRODUCTION

The mature human mammary gland is a compound tubuloalveolar structure composed of milk-secreting polarized epithelial cells surrounded by myoepithelial cells, and the two-layered tissue organization is surrounded by a basement membrane [1]. During the past few decades, studying the physiology of the mammary gland in rodent models has greatly promoted our knowledge about hormone action [2], gene regulation [1, 3], and stem cell biology [4]. However, understanding the developmental process of human mammary glands through the knowledge gained from animal models is not ideal. The mammary glands in rodents and humans are different in several aspects such as anatomical location, histological composition, and gene expression profiles [5, 6]. Furthermore, the whole-animal experiments make it difficult to separate and analyze molecular events at the cellular level [7]. Cultured immortalized human mammary cell lines, such as MCF-10A, have been widely used as *in vitro* models to study the mechanisms that dictate mammary epithelial biology. However, whether these cells serve as appropriate *in vitro* models for human mammary epithelial cells has recently been challenged [8]. Studies have suggested that continuous cell lines exhibit increased lineage-restricted profiles that fail to truly represent the intratumoral heterogeneity of individual breast tissues [9]. For example, normal breast cell lines demonstrate loss of EpCAM^+^CD49f^−^ and EpCAM^+^CD24^+^CD49f^+^ populations compared to primary breast epithelial cells isolated from reduction mammoplasty. Furthermore, although they retain features of bipotent progenitor cells, mammary cell lines such as MCF-10A and HME I/II are unable to differentiate into mature luminal breast epithelial cells [9]. Therefore, *in vitro* models that better recapitulate the physiologically relevant heterogeneity of the epithelial cells of human mammary gland tissue are desired.

Primary epithelial cells derived directly from human mammary glands provide a tissue-specific model, but comes with limitations such as a short life span in conventional tissue culture conditions [10]. A recently established method known as “conditional reprogramming” showed that irradiated fibroblast feeder cells or feeder cell-conditioned medium, together with a Rho-associated kinase (ROCK) inhibitor (Y-27632), can induce rapid and inexhaustible *in vitro* proliferation of primary epithelial cells from normal and malignant tissues from breast, prostate, and lung [11–14]. Moreover, the effects of ROCK inhibitor are completely reversible. Upon removal of ROCK inhibitor, conditional reprogramming cells stop proliferating and turn into terminally differentiated cells [11]. This conditional reprogramming approach has facilitated the development of patient-specific disease models such as non-small cell lung cancer (NSCLC) [15] and ductal carcinoma *in situ* (DCIS) [16] and paved the way for future personalized medicine. For example, forty-eight resistant NSCLC cell lines were successfully generated from tumor tissues of lung cancer patients whose disease had progressed while on treatment with epidermal growth factor receptor or anaplastic lymphoma kinase tyrosine kinase inhibitor [15]. Genetic analyses and pharmacological screening of these cell lines have identified multiple effective drug combinations that suggest potential applications for personalized medicine [15]. However, few published studies have systemically assessed the cultured normal mammary epithelial cells in terms of cell type heterogeneity, cell marker expression, and structural arrangement in three-dimensional (3D) culture. Adopting the conditional reprogramming methodology, a recent study developed an *in vitro* model for human DCIS from lumpectomy or mastectomy samples [16]. The established primary DCIS cultures contained both luminal and basal mammary epithelial cells and maintained tissue heterogeneity [16]. Lack of estrogen receptor-α (ERα) expression was found in primary and TERT-immortalized human mammary epithelial cells (hMECs) [17–19], as well as commonly used normal breast cell lines such as HMT-3522, MCF10A, and 184B5 cells [20–22]. Similarly, ERα expression was lost or undetected in the *in vitro* culture derived from the ERα-positive DCIS tissue [16]. Epithelial cells are known to have an inherent ability to self-organize into complex tissue structures in a 3D system [23, 24], but the study in DCIS did not show whether the conditionally reprogrammed mammary epithelial cells can form defined structures in 3D culture conditions. Another study compared the percentage of stem/progenitor/mature cells among conditionally reprogrammed epithelial cells derived from more than 60 breast specimens, however information about the ERα status and the 3D structure of these cultures was not reported [25].

Our long-term goal is to develop an *in vitro* model to study normal human mammary cell and tissue function and regulation. In working towards this goal, we exploited the conditional reprogramming method to culture primary human mammary cells from normal prophylactic tissues in both 2D and 3D culture conditions. We have demonstrated the ability of the culture to maintain heterogeneity in both luminal and myoepithelial cellular characteristics. ERα expression and response of primary mammary cells to estrogen stimulation were observed in short-term cultures. In addition, primary cultures spontaneously organized into distinct 3D organizations that are composed of cells expressing different epithelial markers. These results suggest that the conditionally reprogrammed cells might serve as a relevant model for the study of normal mammary cell biology.

## RESULTS

### Morphologies of mammary epithelial cells cultured by conditional reprogramming

**Figure 1:**
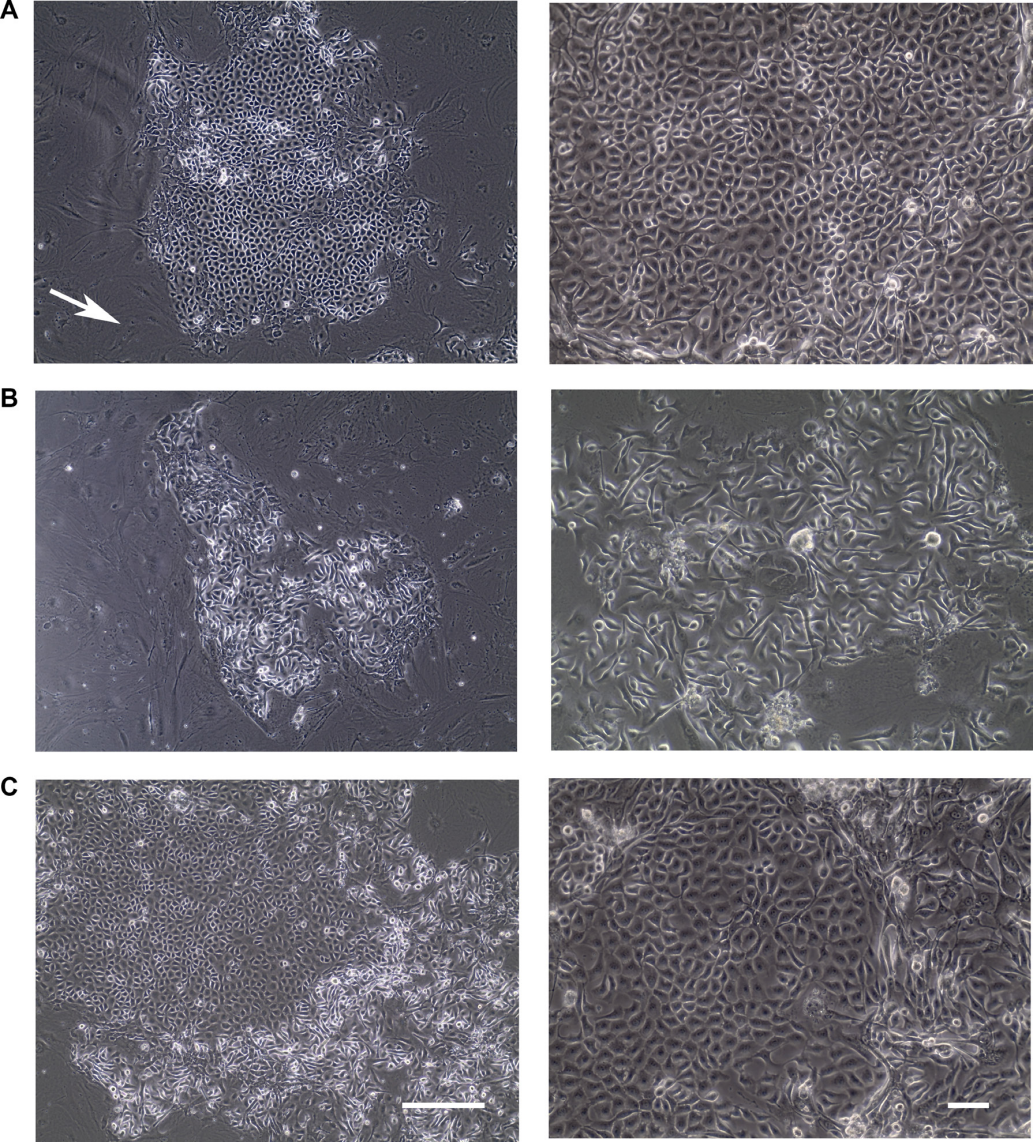
Colonies formed from normal human epithelial cells under conditional reprogramming condition (**A–C**) Representative phase contrast pictures of colonies with various morphologies within a week of culture were shown. Feeder cells were indicated by white arrow. Bars: 200 μm. Original magnification: x100 (left); x200 (right).

Primary epithelial cells grow for a finite life span and eventually senesce [10, 26]. Of note, those cells usually lose phenotypic heterogeneity during *in vitro* culture, exemplified by the gradual conversion of human mammary luminal epithelial cells to myoepithelial cells when cultured *in vitro* [27]. Therefore, we first investigated if epithelial cells isolated from normal human mammary tissue could be grown and expanded using the recently established conditional reprogramming method [12]. When isolated mammary gland epithelial cells from four tissue samples were plated on feeder cells, multiple colonies were formed a week later. Most colonies were composed of cells exhibiting a tightly packed cobblestone appearance (Figure 1A). A smaller number of colonies were composed of scattered, thin, spindle-shaped cells (Figure 1B). Even less frequently, colonies were found to comprise a mixed cell population with cobblestone-like cells in the center and spindle-shaped cells at the edge (Figure 1C). We observed none or few mammary fibroblasts in the culture, possibly because this culture condition favors the growth of epithelial cells [12]. With continuous passaging, a few cells appeared slightly enlarged compared to the cells in earlier passages, but the cultures still retained their original epithelial traits in general (data not shown), suggesting that the culture conditions preserved the cellular or morphological heterogeneity. These results demonstrate that the conditional reprogramming method allows primary mammary epithelial cells to be cultured *in vitro* with an extended life span.

### Cultured primary mammary cells express epithelial cell markers

**Figure 2:**
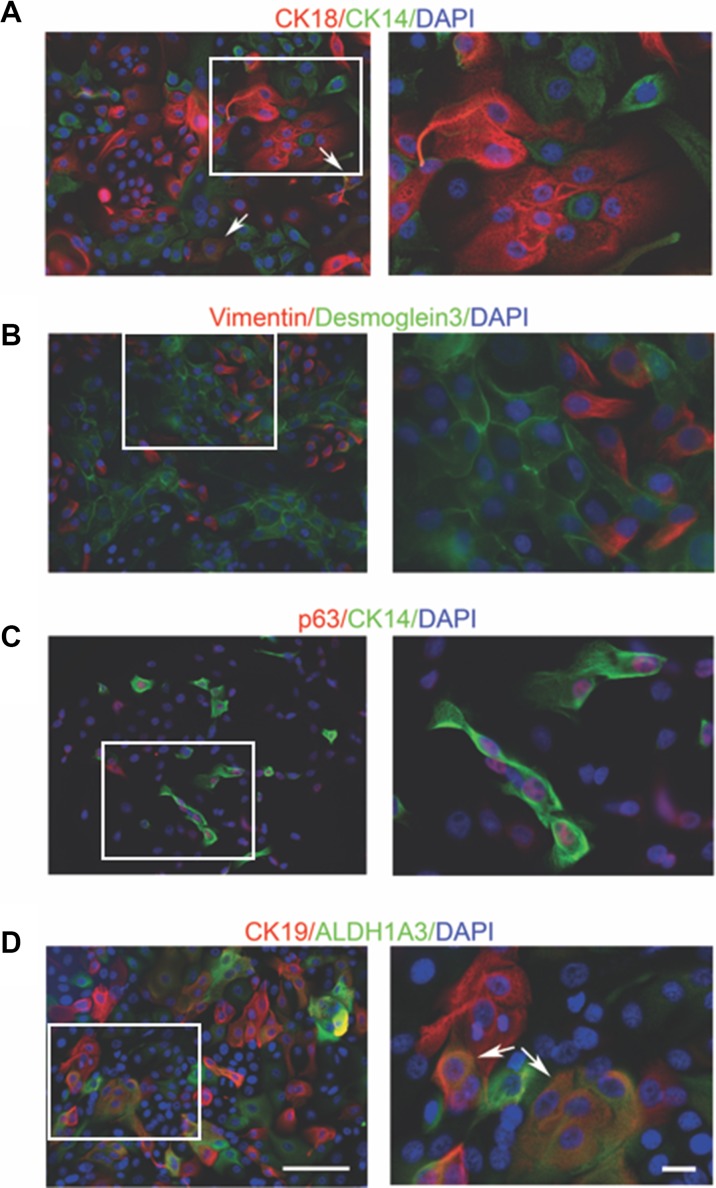
Immunofluorescent staining of mammary cell markers in conditional reprogrammed cells in early passage The expression of (**A–C**) luminal and myoepithelial markers, (**D**) mature luminal and stem/progenitor cell markers was measured. All the cultures used in the immunofluorescent staining were from passage 4 (14–261). White arrows indicate the cells co-stained with two markers. The area on the left marked by the white border is shown by the magnified image on the right. Bars: 100μm. Original magnification: x200 (left); x400 (right).

We next characterized the epithelial identity of cultured primary cells using immunofluorescence. As shown in Figure 2A, mammary cells were either positive for cytokeratin 18 (CK18), a marker of luminal epithelial cells [28], or cytokeratin 14 (CK14), a marker of myoepithelial cells [29]. Notably, a few cells (arrow-labeled) were positive for both markers. Desmoglein3 is an important component of the desmosome, an intercellular junction of epithelia [30, 31]. Its expression was only detected at the interaction surface of cobblestone-shaped epithelial cells. while expression of vimentin, a myoepithelial marker [29], was absent in these cells (Figure 2B). Conversely, desmoglein 3 was not detected in vimentin^+^ epithelial cells (Figure 2B). CK14 and p63 are both myoepithelial markers with different subcellular locations [32]. As expected, co-staining of cytoplasmic CK14 and nuclear p63 was observed (Figure 2C).

Aldehyde dehydrogenase (ALDH) activity is commonly used to identify stem/progenitor cells [33] and this activity can be attributed to ALDH1A3 in stem cells [34]. ALDH1A3 expression was detected in a subgroup of cells and some ALDH1A3^+^ cells co-expressed cytokeratin 19 (CK19) (Figure 2D). CK19 is a luminal marker [3] and loss of CK19 has been reported during *in vitro* culture of primary epithelial cells [35]. Interestingly, we observed evident CK19 staining in our culture and a subset of CK19^+^ cells harbored ALDH1A3 expression. A combination of CD49f (α6 integrin) and epithelial cell adhesion molecule (EpCAM) is widely used to define cells within the luminal and myoepithelial lineages from normal human breast tissue [9, 36]. We then carried out the flow cytometry (FACS) analysis to compare cell subpopulations with different CD49f and EpCAM expression status. As shown in Supplementary Figure 1A and 1B, four cell populations were identified. The CD49f+/EpCAM+ phenotype was found to be the major fraction, followed by the CD49f+/EpCAM- and CD49f-/EpCAM+ populations (Supplementary Table 1), which is consistent with an earlier report [25]. The heterogeneous CD49f and EpCAM expression in the total cell population was also confirmed by immunofluorescent staining (Supplementary Figure 1C).

### Cultured primary cells retain ERα expression and respond to estrogen stimulus

**Figure 3:**
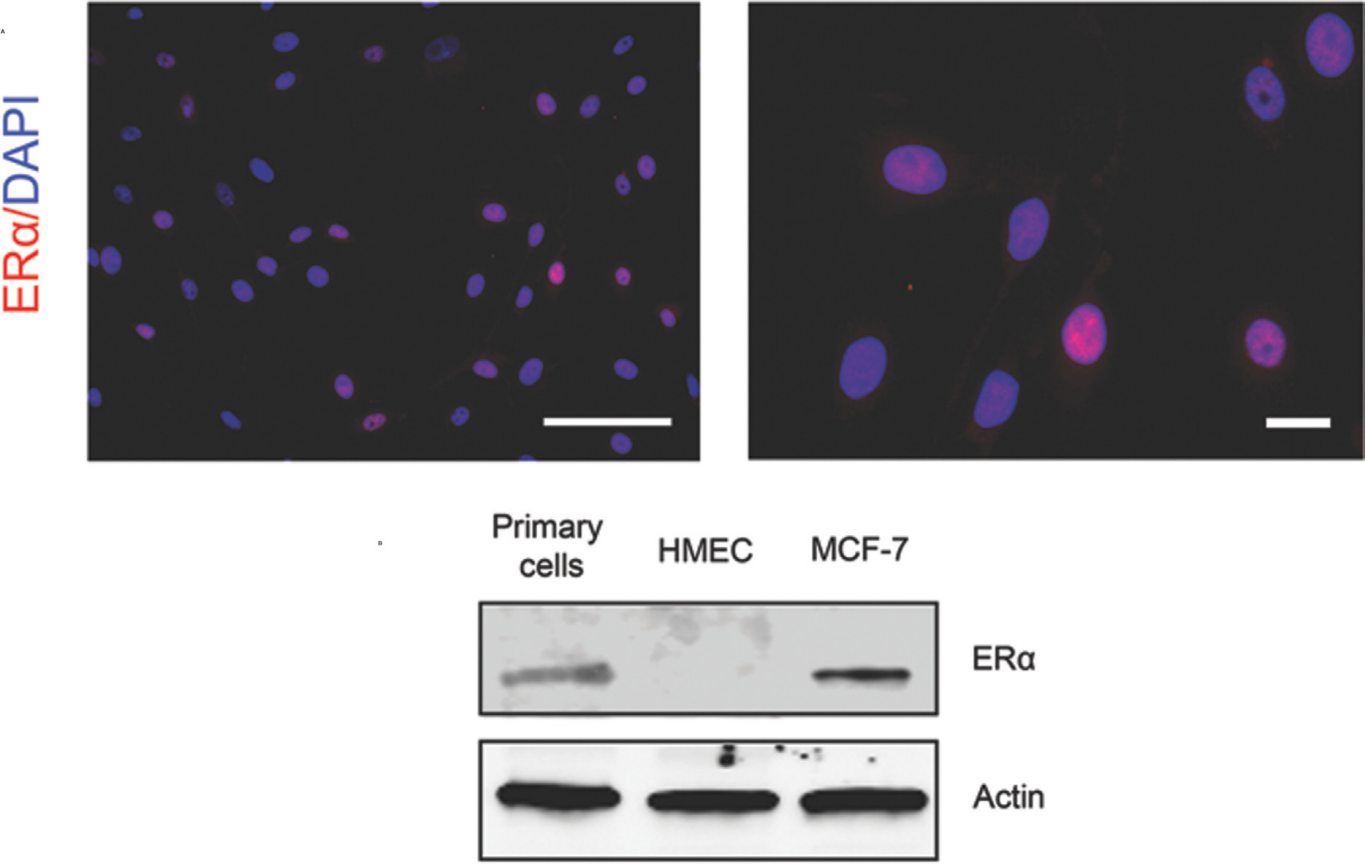
Expression of ERα in the cultured primary mammary epithelial cells (Top) ERα expression was examined by immunofluorescent staining. Secondary antibodies conjugated with AF594 (red) were used to visualize the signal. (Bottom) Western blotting analysis of ERα protein expression in the primary mammary epithelial cells. MCF7 and hMEC cells were used as positive and negative controls. Bars: 100 μm. Original magnification: x200 (left); x400 (right).

**Figure 4:**
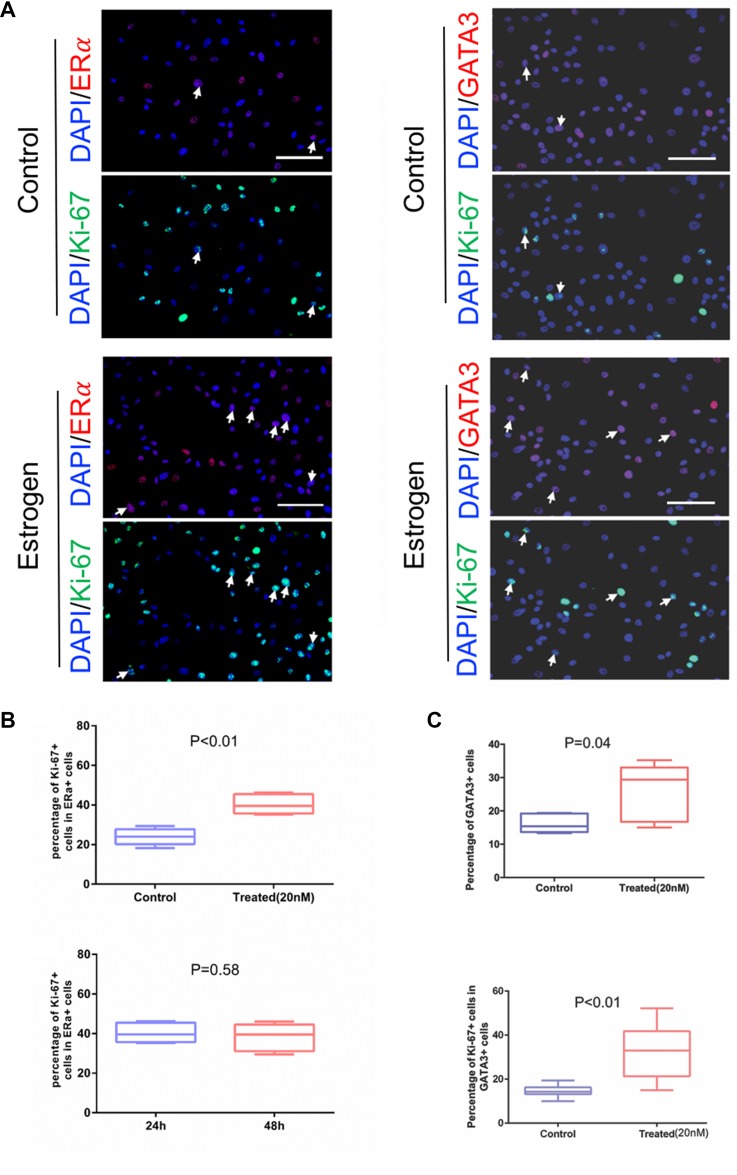
Proliferative response of the primary cultured mammary epithelial cells to estrogen (**A**) ERα/GATA3 and Ki-67 expression were measured by immunofluorescence, AF594 (red) and AF488 (green)-conjugated secondary antibodies were used respectively to visualize the signals. Bottom panel: treated with estradiol, top panel: vehicle as control. The images of Ki-67 and ERα or GATA3 staining were taken from the same field of view. White arrows indicate proliferating ERα^+^/GATA3 cells. (**B**) Five randomly selected view fields were used to count ERα^+^/Ki-67^+^ and total ERα^+^ cells, calculated the proportion of ERα^+^Ki67^+^ in ERα^+^ cells were plotted. Unpaired *t* test was used to analyze the results, *P* < 0.01. Cultured cells were treated with estradiol for 24 hours or 48 hours. Difference in incubated time with estradiol, the percentage of ERα^+^ proliferating cells were analyzed, *P* = 0.58. (**C**) Five randomly selected view fields were used to count GATA3^+^/Ki-67^+^ and total GATA3^+^ cells, calculated the proportion of GATA3^+^ cells after treated with estradiol or vehicle, unpaired *t* test was used to analyze the results, *P* = 0.04. Furthermore, calculated the proportion of GATA3^+^/Ki-67^+^ in GATA3^+^ cells were plotted and unpaired *t* test was used to analyze the results, *P* < 0.01. Bars: 100 μm. Original magnification: x200.

Previous studies have shown consistent lack of ERα expression in *in vitro* cultures of immortalized and primary normal mammary cells [17–19, 21, 37]. Here, we tested our method to see if ERα expression is preserved. As shown in (Figure 3, top), strong nuclear staining of ERα protein was observed in a subset of cells. The test was repeated in four cultured samples with the percentage of ERα^+^ cells ranging from 5% to 30% initially (Supplementary Table 1). The percentage of ERα^+^ cells is comparable with a previous finding that ERα immunoreactivity was found in the nuclei of 5% to 28% of epithelial cells in acini and interlobular ducts of normal human mammary glands [38]. ERα signal had a moderate decrease in the early passages, and its drop was more prominent in later passages. We further performed immunoblotting analysis to confirm the expression of ERα in the cultures (Figure 3, bottom). In order to determine if the retained ERα^+^ cells respond to estradiol stimulation, we measured the proliferation of cultured cells when treated with estradiol relative to vehicle control (Figure 4A, left panel, white arrows) using ERα and Ki-67 co-staining. We observed that the percentage of ERα^+^/Ki-67^+^ was increased in the ERα^+^ pool (*P* < 0.01, Figure 4B, top panel), indicating that the proliferation of ERα^+^ cells was stimulated by estradiol stimulation. This observation was also showed in 48h estradiol treatment. But the percentile of Ki-67^+^ cells in ERα^+^ population in 24-h and 48-h treatment did not show differences (*P* = 0.58, Figure 4B, bottom panel). To consolidate the study on the cell response to estradiol, we performed immunostaining of GATA3 and Ki-67 in primary cells (Figure 4A, right panel, white arrows). GATA3 is a key luminal transcription factor. It co-expresses with its direct target ERα and forms a positive cross-regulatory loop with ERα in which ER directly stimulates the transcription of the GATA3 gene [39–41]. As demonstrated in Figure 4C, we observed more GATA3^+^ and Ki-67^+^/GATA3^+^ cells when the primary culture was treated with estrogen compared with vehicle control (*P* < 0.01).

### Primary cells organize into heterogeneous structures in 3D culture

**Figure 5:**
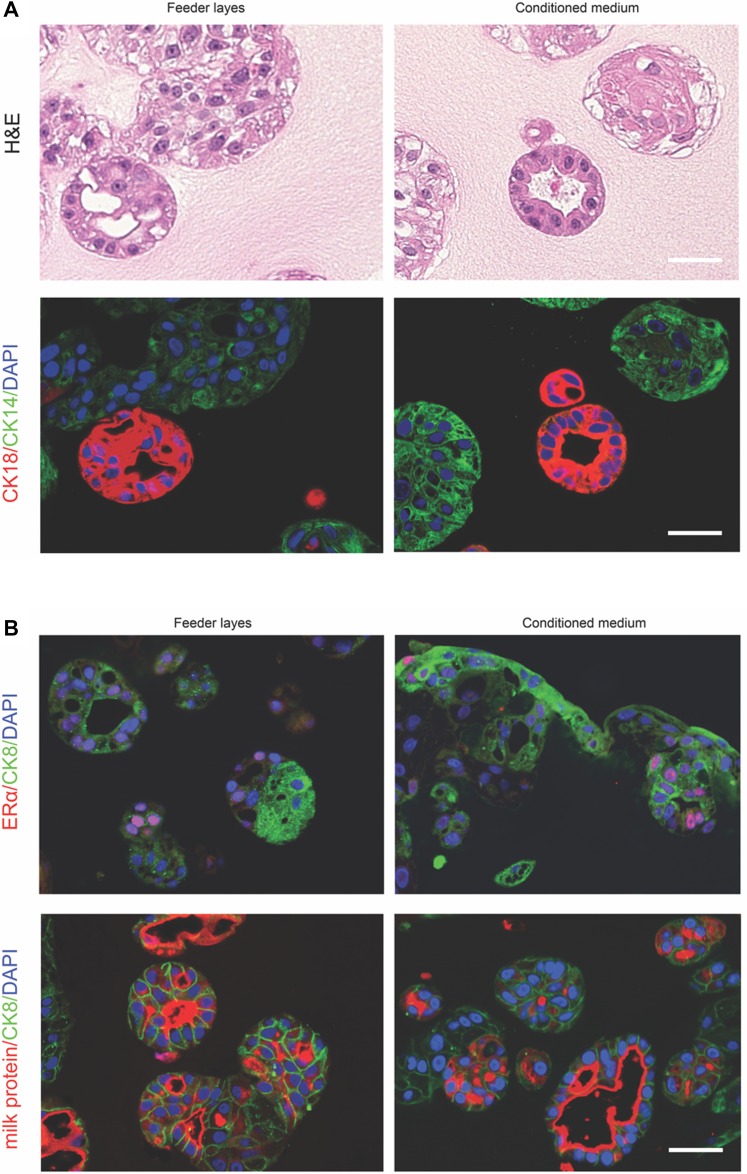
Conditional reprogrammed epithelial cells in 3D culture (**A**, top panel), haematoxylin and eosin-stained section of the structures produced in the 3D culture. (A, bottom panel), cellular expression of CK14 and CK18. (**B**, top panel), ERα and CK8 immunofluorescence staining. (B, bottom panel), milk protein and CK8 immunofluorescence staining. Primary cells (passage 4) were cultured with feeder cells in fresh medium (left column) or in feeder cell-conditioned medium (right column). Bars: 50 μm. Original magnification: x400.

It has been reported that when plated into Matrigel, luminal progenitor cells (CD49f^+^EpCAM^high^) gave rise to uniformly spherical acinar structures composed of single-layer cuboidal epithelial cells expressing luminal markers [36]. In contrast, structures derived from basal or bipotent progenitor cells (CD49f^+^EpCAM^−/low^) were solid, irregular-shaped colonies that exhibit myoepithelial markers [36]. Thus, we explored if our primary culture comprised of luminal and myoepithelial cell populations would be able to generate distinct colonies in 3D culture. To this end, we grew the conditionally reprogrammed epithelial cells (passage 4) in Matrigel/Collagen I gel with irradiated feeder cells in fresh medium (Figure 5, left) or in feeder cell-conditioned medium only (Figure 5, right) for 2 weeks. Two types of structures formed in the mixed gel: round ductal structures with a single layer of cells enclosing a lumen and poorly organized ball-like colonies (Figure 5A, top panel). Notably, both conditioned medium and feeder cells yielded similar results that are in line with the previous finding [13]. Furthermore, immunofluorescence staining showed that the lumen-containing structure was composed of cells expressing luminal lineage markers CK18. In contrast, the ball-shaped structure only consisted of cells displaying basal markers CK14 (Figure 5A, bottom panel). Consistent with this result, ERα staining was found in a subset of CK8^+^ cells (Figure 5B, top panel). We also detected strong expression of milk protein in the lumen enclosed by CK8^+^ cells (Figure 5B, bottom panel). Normal human mammary tissue was used as a positive control for milk protein staining (Supplementary Figure 2). Taken together, these data suggest that our culture maintains the ability to spontaneously organize into distinct structures.

## DISCUSSION

Conditional reprogramming method has been used to expand primary epithelial cells from different normal human tissues [12]. In this study, we further investigated the characteristics of reprogrammed mammary epithelial cells and demonstrated that these cells retain many important features associated with cells from mammary tissues. The cultures contain cells expressing markers associated with luminal and myoepithelial cells. Some of the conditionally reprogrammed cells expressed both luminal and myoepithelial markers. Their cell identity is not clear. A gradual decrease in expression of CK19 during *in vitro* culture of primary epithelial cells has been reported as evidence of trans-differentiation from epithelial to mesenchymal phenotype [35]. The sustained CK19 staining in our culture indicates this model is able to preserve certain characteristics inherited from primary tissues within early passages. EpCAM and CD49f are widely used to define subpopulations of mammary epithelial cells. Luminal progenitors are known to exhibit a CD49f^+^EpCAM^high^ profile while myoepithelial cells and putative bipotent progenitor cells are defined by the CD49f^+^EpCAM^−/low^ phenotype [9, 36]. Previous publications showed normal breast cell lines lose CD49f^−^EpCAM^high^ and CD49f^+^CD24^+^EpCAM^high^ cells. However, in our culture, we found four different subpopulations characterized by different expression levels of EpCAM and CD49f, including CD49f^+^EpCAM^high^ and CD49f^+^EpCAM^−/low^ cells. Cells with a CD49f^−^EpCAM^high^ profile, characteristic of mature luminal cells, were also detected. Therefore, we consider the conditional reprogramming method to retain the complex heterogeneity of mammary epithelial cells.

Under a 3D Matrigel/collagen I gel condition, we were able to obtain structures similar to those generated by CD49f^+^EpCAM^high^ or CD49f^+^EpCAM^−/low^ cells, which have been considered as progenitor populations. Each structure could be formed by either the same types of progenitor cells or clumps of different progenitor cells, providing a possible explanation to the mixed structures found in a few colonies. Alternatively, those mixed structures could be generated from bipotent progenitor cells [42]. Earlier studies established mammary ductal structures from freshly isolated mammary organoids by seeding them onto hydrogel scaffolds [23] or transplanting them into mice [43]. However, these methods relied on the original structural arrangement of different mammary cells, limiting the scope of the methods to studying regulation of cell specification, function, and differentiation. The conditional reprogramming method, on the other hand, allows *in vitro* propagation of epithelial cells, and preserves the progenitor-like populations and the structure-forming ability. Due to the rarity of repopulating cells among the whole epithelial cell population (about 1 per 1 × 10^3^ epithelial cells), it requires at least 1 × 10^5^ primary epithelial cells for the mice transplantation [43]. In contrast, conditional reprogramming does not rely on the subset of progenitor cells, but rather converts mature epithelial cells into those possessing stem-like properties and proliferating inexhaustibly *in vitro*, which enables expanding the culture from a very limited number of epithelial cells [12].

Previous studies have shown absence of ERα expression in *in vitro* cultures of mammary cells. For example, ERα expression was lost in the reprogrammed cells derived from human DCIS tissues [16]. A recent study isolated ERα+ cells from fresh normal hMECs and tracked ER loss in these cells during culture [20]. They demonstrated that TGFβR2 inhibitor was able to induce the restoration of ERα expression in cultured HMEC [20]. It is worth mentioning that we detected nuclear ERα staining in our 2D and 3D cultures and these ERα^+^ or GATA3^+^ cells proliferated in response to estrogen stimulation. We found that the ERα expression was retained in short-term culture. It is not clear whether ERα expression will be completely lost in later culture passages.

In conclusion, we have shown that the conditional reprogramming methodology allows *in vitro* generation of heterogeneous culture from normal human mammary tissue. Importantly, ERα expression could be detected in these cultures and estrogen activates ERα function. This primary culture technique supports the development of milk-producing cells and organization of distinct 3D organoid structures. This approach may provide an invaluable tool to study mammary cell function and factors involved in malignant transformation. Further studies are warranted to test whether conditionally reprogrammed cells maintain their function *in vivo*.

## MATERIALS AND METHODS

### Tissue source information

The protocol for culturing primary mammary epithelial cells was adapted from recently published methods [12–14]. This study was approved by the Institutional Review Board (IRB) at Cedars-Sinai Medical Center.

### Mammary epithelial cell isolation and culture

Normal human breast tissues were obtained from prophylactic surgeries with written informed consent. In brief, fresh mammary gland tissues were minced into 1 mm^2^ pieces and dissociated in collagenease and hyaluronidase mixture (Stemcell Technologies, Vancouver, BC, Canada) overnight at 37°C. The tissue enzyme mixture was then filtrated through a nylon strainer and the flow-through fractions were collected and centrifuged at 1200 rpm for 10 minutes to obtain the pellets. Accumax (Innovative Cell Technologies, San Diego, CA) was used to further dissociate the organoids to obtain mostly single cells. Cells were re-suspended in F-medium. F-medium contains Dulbecco’s Modification of Eagle’s Medium /Ham’s F-12 50/50 with L-glutamine (DMEM/F12; 1:1) (Corning, Corning, NY), 5% FBS, 0.4 μg/mL hydrocortisone, 5 μg/mL insulin, 8.4 ng/mL cholera toxin, 10 ng/mL epidermal growth factor (all, Sigma, St. Louis, MO), and 24 μg/mL adenine with addition of 10 μM Y-27632 (Enzo Life Sciences, Farmingdale, NY). The cells were then plated on a layer of 50% confluent irradiated or mitomycin-treated 3T3-J2 feeder cells for further expansion. All cultures were maintained at 37°C and 5% carbon dioxide. The initial colony expansion took 1–2 weeks to reach 80–90% confluence in a well of a 6-well plate and cells were split at 1:10 for later passages. Once cells reached 80–90% confluence within 4–6 days, cells were passaged with Accutase (Innovative Cell Technologies) to avoid the harsh enzymatic condition.

### Immunofluorescent staining

For 2D culture, cells were plated in 8-well chamber slides and kept in conditioned medium [13] (three volumes of feeder cell-conditioned F-medium mixed with one volume of fresh F-medium supplemented with 5 μM Y-27632) to grow in a monolayer and then fixed with 4% paraformaldehyde for 15 minutes, permeabilized with 0.5% Triton X-100 for 15 minutes, and blocked with 5% bovine serum albumin for one hour at room temperature. After blocking, cells were incubated overnight at 4°C with primary antibodies (Supplementary Table 2). Alexa Fluor (AF) 594-(red, Molecular Probes, Eugene, OR) or AF488-conjugated secondary antibody (green, Molecular Probes) were used to visualize the signal. Following three washes with PBS, slides were mounted with the VECTASHIELD mounting medium with DAPI (Vector Laboratories, Burlingame, CA). The fluorescence images were taken using the EVOS FL Auto fluorescence microscope (Life Technologies, Carlsbad, CA).

### Estrogen-induced proliferation assay

Primary mammary epithelial cells (passage 3) were plated into 4 wells of an 8-well chamber slide with 80% confluence and cultured overnight in F-medium. Estradiol (20 nM) was added into 2 wells and the vehicle was added to the other two wells. The cultures were incubated for 24 hours and 48 hours after Estrogen stimulation and then subject to fixation. Co-staining of ERα or GATA3 and Ki-67 were detected by immunofluorescence. Ki67, a proliferation marker [44–46], was used to analyze the proliferate ability of ERα^+^ or GATA3^+^ cells in our estradiol-induced proliferation assay. AF488 (green)-conjugated secondary antibody was used to visualize the Ki-67 signal, while AF594 (red) was used to visualize the ERα or GATA3 signal. Five randomly selected fields from each well were analyzed to calculate the proportion of ERα^+^Ki67^+^ cells in the total ERα^+^ pool, or calculate the proportion of GATA3^+^Ki-67^+^ cells in the total GATA3^+^ pool. An unpaired *t* test was used to analyze the difference between the estradiol treated group and the vehicle control group.

### 3D culture

3D “cell-embedded” culture was performed as previously reported [8, 47]. In brief, cell pellets were mixed with growth factor-reduced Matrigel (Sigma, St. Louis, MO)/collagen I (4 mg/mL, Advanced BioMatrix, Carlsbad, CA) gel (3:1 in volume) and seeded in low attachment 24-well plates (70 μL/well, Nunclon Delta Surface plate, Thermo Fisher, Canoga Park, CA). Approximately 0.8×10^5^ cells were seeded into each well of a 24-well plate for culture. The final concentration of collagen I in the mixed gel was 1 mg/mL [48]. In order to compare the effects of conditioned medium and feeder cells, two culture conditions were applied in the following steps: (1), the solidified gels were detached from the wells and kept floating in the conditioned medium during culture. (2), the solidified gels were detached and transferred to a different well pre-coated with feeder cells and fresh F-medium was added. For both conditions, the medium was changed every 2 to 3 days. After 2 weeks of growth, cells were fixed with 4% paraformaldehyde, washed with PBS, and embedded in Specimen Processing Gel (Histogel, American MasterTech Scientific, Lodi, CA). Samples were then processed and embedded in paraffin. Blocks were cut into 4-μm thick slides for staining. Slides were deparaffinized and rehydrated by xylene and gradient ethanol, respectively. An antigen retrieval method by microwave pretreatment and 0.01 M sodium citrate buffer (PH6) (Vector laboratories, Burlingame, CA) was used for the following staining.

### Flow cytometry (FACS)

Cells (passage 4 unless otherwise denoted) derived from four patients (14–261, 14–313, 16–025, 4700wt (passage 5)) were isolated, and approximately 2 × 10^5^ cells were suspended in FACS buffer (1×PBS, 1% BSA) and incubated with antibodies at 4°C for 30 minutes. EpCAM-FITC (5 μL per reaction, #347197, BD Biosciences, San Jose, CA) and CD49f (α6 integrin)-AF647 (1 μL per reaction, #562494, BD Biosciences) antibodies were used in FACS. Samples were analyzed by LSRII Flow Cytometer (BD Biosciences). Approximately 20,000 cells were collected for each sample. Data was analyzed using FlowJo Version 7.6.5 software.

### Immunoblotting analysis

Total protein extraction and immunoblotting were performed using methods described previously [49]. Primary antibody against ERα was used to determine the level of ERα protein expression (Supplementary Table 1). β-actin (1:200, Santa Cruz) was used as a loading control for total protein.

## SUPPLEMENTARY MATERIALS FIGURES AND TABLES



## Abbreviations

2D: two-dimensional
3D: three-dimensional
ROCK: rho-associated kinase
NSCLC: non-small cell lung cancer
DCIS: ductal carcinoma *in situ*
ERα: estrogen receptor-α
hMECs: human mammary epithelial cells
CK18: cytokeratin 18
CK14: cytokeratin 14
ALDH: Aldehyde dehydrogenase
CK19: cytokeratin 19
EpCAM: epithelial cell adhesion molecule
FACS: flow cytometry
