# Supplementary material for: Characterization of primary human mammary epithelial cells isolated and propagated by conditional reprogrammed cell culture
Source: Oncotarget. 2017 Dec 22;9(14):11503–14. doi: 10.18632/oncotarget.23817 (PMC5837767; doi:10.18632/oncotarget.23817)
Supplement: Supplementary file 1 [file oncotarget-09-11503-s001.pdf]

Characterization of primary human mammary epithelial cells isolated and propagated by conditional reprogrammed cell culture

SUPPLEMENTARY MATERIALS

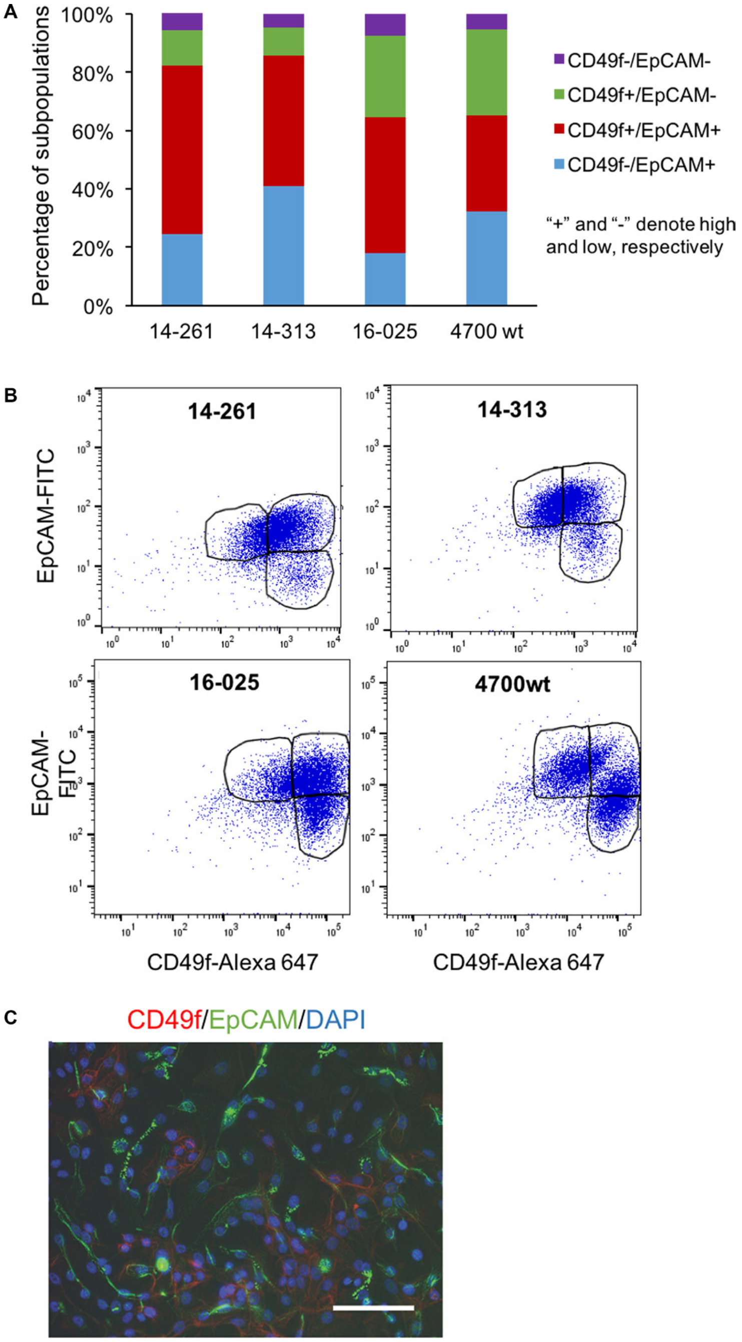

Supplementary Figure 1: CD49f/EpCAM expression of primary human mammary cells. Primary human mammary cells (samples 14–261, 14–313, 16–025, 4700 wt) were extracted from breast tissue and stained for EpCAM-FITC (green) and CD49f-AF647 (red). (**A**) Graphical representation of the percentages of CD49f-/EpCAM+, CD49f+/EpCAM+, CD49f+/EpCAM- and CD49f-/EpCAM- cells (see Supplementary Table S1 for quantification). (**B**) Flow cytometry analysis of CD49f-/EpCAM+, CD49f+/EpCAM+, CD49f+/EpCAM- and CD49f-/EpCAM- cells from the four samples. Circles were drawn to represent and quantify the CD49f-/EpCAM+ (top left), CD49f+/EpCAM+ (top right), and CD49f+/EpCAM- (bottom right) regions. (**C**) Immunofluorescence staining of fixed primary human mammary cells for EpCAM, CD49f, and the nucleus. "+" and "-" denote "high" and "low" expression, respectively.

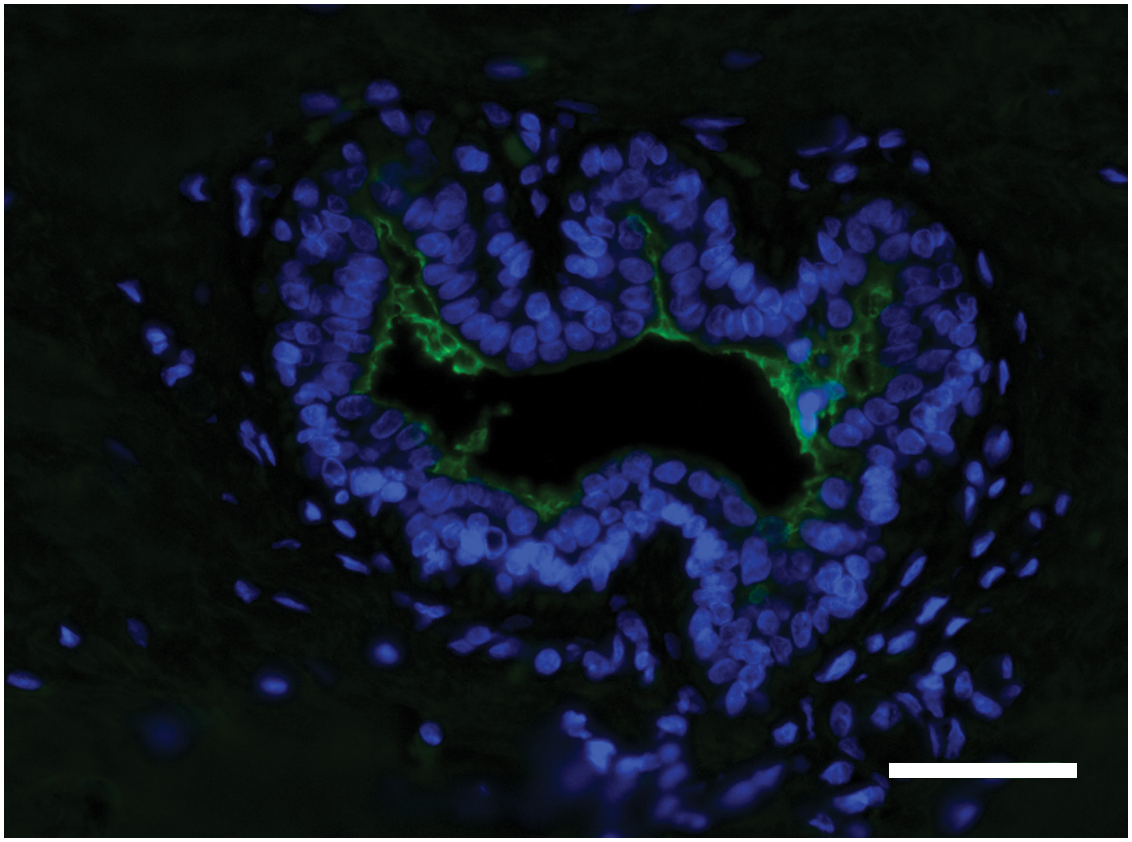

Supplementary Figure 2: Milk protein expression in normal human breast tissue. Milk protein expression in human breast tissue was examined by immunofluorescent staining. Secondary antibody conjugated with AF488 (green) was used to visualize the signal. DAPI was used for nuclear staining. Bars: 50 µm. Original magnification: x400.

Supplementary Table 1: ERα status and CD49f/EpCAM profiles in tested samples

Sample	ERα+	CD49f-/EpCAM+	CD49f+/EpCAM+	CD49f+/EpCAM-	CD49f-/EpCAM-
14-261	27.0%	24.3%	58.1%	12.0%	7.5%
14-313	30.0%	40.8%	44.8%	9.81%	4.59%
16-025	< 5%	17.9%	46.6%	28.0%	7.5%
4700wt	< 5%	32.1%	33.1%	29.4%	5.4%

Note: $ER\alpha^+$ cells in 16-025 and 4700wt were not detected by staining after passage 4.

Supplementary Table 2: List of primary antibodies used in this study

Primary antibody	Marker for	Source	Dilution
ERα	luminal	Biocare	1:100
ERα	luminal	Santa Cruz	1:200
GATA3	luminal	Santa Cruz	1:200
PR	luminal	Biocare	1:100
CK18	luminal	Abcam	1:200
CK14	myoepithelial	Biocare	1:200
P63	myoepithelial	Biocare	1:200
vimentin	myoepithelial	Biocare	1:200
CK19	luminal	Abcam	1:200
Desmoglein3	luminal	Biocare	1:100
EpCAM	epithelial	Abcam	1:200
CD49f	Stem/progenitor	R&D	1:200
ALDH1A3	Stem/progenitor	Santa Cruz	1:200
Ki-67	proliferation	Abcam	1:500
Milk protein	Human milk	Nordic Immunological Laboratories	1:500
